# The Effect of Variola in the Mother upon the Fœtus in Utero

**Published:** 1866-03

**Authors:** E. Griffin

**Affiliations:** Surgeon of the City Small Pox Hospital, of Atlanta, Georgia


### ARTICLE III.

*The Effect of Variola in the Mother upon the Fœtus in Utero.* By E. Griffin, M. D., Surgeon of the City Small Pox Hospital, of Atlanta, Georgia.

That the child in utero, at any period of pregnancy, may be impressed by small pox, has been established beyond all doubt. It has not, however, been so clearly ascertained at what period of the disease in the mother the child receives the contagion. Cases have been witnessed, in which the evidences seemed to justify the opinion that the disease was contracted simultaneously by the mother and child; others, in which the child probably contracted the disease about the period of febrile symptoms in the mother, preceding the eruption; and again, others, in which the fœtus evidently imbibed the contagion at the first appearance of the eruption, and even at a still later period. The time of incubation being pretty uniform, there is no great difficulty in determining the period at which the disease was contracted, when the first appearance of the eruption is witnessed, or from their character or condition the length of time which the pustules have existed, can be estimated.

In 1863, while in charge of the Fulton county small pox hospital, two cases of premature delivery in females affected with small-pox, came under my care. One of them was an inmate of the hospital, and under my immediate supervision; the other, though in a private family, was under my observation and treatment. In both, the delivery occurred about the tenth day of the eruption, when suppuration had commenced in the pustules, and at about the fourth and sixth month respectively, of utero-gestation. In each case the eruption of variola was found upon the child, exhibiting evidences of the same stage of advancement as that found upon the mother, and in one case pretty nearly the same amount of vescicles, while in the other the eruption was not so abundant as on the mother.

These facts afford conclusive evidence that the mother may contract the disease and communicate to the foetus the contagion directly, through the intimate connection which exists between them. And the fact that in more advanced stages of pregnancy we sometimes find that the child does not contract the disease until about the time the eruption appears on the mother, only proves that the disease may be contracted by the fœtus in the ordinary way, after the mother has passed through the period of incubation, as well as simultaneously with the mother, from the same external influences conveyed through her to the child.

A case of delivery at the full period of utero-gestation occurred recently with a female, who had ten days previously all the symptoms preceding the eruption of small pox, which after remaining the usual time of three or four days, disappeared without the eruption, and left the woman comparatively well up to the time of her confinement in labor. The child, healthy and vigorous, remained lively for about a week, when fever and indisposition to take food, gave evidences of disease. These symptoms continued three or four days, when the eruption of small pox made its appearance in the confluent form. The case is now progressing, with little or no prospect of recovery.

It cannot be said, in this case, that the child contracted the disease after birth, because the time from delivery to the development of the disease, being only six or seven days, was not more than half the usual period of incubation. Neither is it very reasonable to my mind that, if the symptoms alluded to in the mother were those of the development of variola, both could have received the constitutional impression at the same time; for in that event the period of incubation would have been twice the usual length. To date back, from the symptoms of variola in the child, the usual incubation period, it will be found that about the time that symptoms in the mother were manifested attributable to small pox, the period should have commenced. Now whether in the different stages of fœtal development impressions made upon the mother are in a different manner received by the child, is a question, the proper consideration of which may reveal the true explanation of this physiolo-pathological difficulty.

Another case of delivery, eight weeks after the mother had suffered from small pox, came under my notice, in which the child exhibited the appearance of variola cicatrices similar to those found upon the mother. Nothing definite, however, can be determined from this case, inasmuch as nothing in the appearance of the child could be detected by which it could be ascertained with any degree of certainty whether or not the eruption in the mother and child occurred simultaneously.

In regard to the vaccination of the mother giving immunity to the child in utero against variola or vaccinia ever afterwards, I do not feel warranted, from my limited observation on this point, in giving an opinion. Some facts have come under my knowledge which favor the affirmative, yet not in a way to be entirely satisfactory.

It is hoped that this subject will engage the attention of physicians having charge of small pox in pregnant females.

